# Shared Reading as a Community of Practice for Overcoming the Generation Gap and Improving Psychological Safety in Rural Family Medicine Education: A Grounded-Theory Approach

**DOI:** 10.7759/cureus.47331

**Published:** 2023-10-19

**Authors:** Ryuichi Ohta, Takuji Katsube, Chiaki Sano

**Affiliations:** 1 Communiy Care, Unnan City Hospital, Unnan, JPN; 2 Family Medicine, Unnan City Hospital, Unnan, JPN; 3 Community Medicine Management, Shimane University Faculty of Medicine, Izumo, JPN

**Keywords:** generation gap, psychological safety, family medicine, japan, medical education, rural medical education, community of practice, shared reading

## Abstract

Background

A community of practice (CoP) is essential for continuous professional development in family medicine. A CoP in medical education comprises a group of individuals who share common interests and learn and share knowledge, skills, and attitudes. The generation gap between teachers and learners can influence their effective collaboration in family medicine education. To address this issue, shared reading, which involves group discussions on medical texts, effectively promotes intergenerational learning within a CoP. Shared reading is particularly beneficial in rural contexts, where medical resources are scarce, and the generation gap between teachers and learners is wide. This study examines how shared reading facilitates learning and practice among family medicine trainees in rural areas.

Methods

This qualitative study utilized a grounded-theory approach. It involved the participation of eight family medicine residents, five junior residents, and seven medical students from Unnan City Hospital, Japan, in shared reading sessions between August 2022 and March 2023. Semi-structured interviews were conducted with all participants after the sessions.

Results

Data analysis using the grounded-theory approach yielded three themes: facilitating learning and motivation, respectful collaboration with teachers, and developing a relationship that fosters psychological safety. First, through shared reading, the participants had opportunities to learn about medical issues and engage in continual dialogues with colleagues and teachers. Second, the participants felt motivated to apply their newly acquired knowledge at work and collaborate with teachers. Third, they acquired self-regulated learning skills by adapting their motivations to their interests and experiences. Increased interaction between participants and teachers during the sessions helped mitigate the generation gap and enhanced psychological safety.

Conclusions

Shared reading effectively promotes continuous learning and motivates medical learners to apply their knowledge and collaborate with teachers. It facilitates the development of self-regulated learning skills, helps mitigate the generation gap, and enhances psychological safety among educators and learners in rural medical education.

## Introduction

A community of practice (CoP) is pertinent for continuous professional development in family medicine [1]. In medical education, a CoP is a group of individuals who share a common interest and learn and engage in sharing knowledge, skills, and attitudes [2]. CoPs are formed based on specific medical topics with members from different backgrounds. Family medicine practitioners are required to continuously improve their knowledge, skills, and attitudes to address various biopsychosocial issues in patients [3]. CoPs offer an avenue for family physicians to discuss these issues, leading to better patient care [4]. They can take different forms, including online communities, local or regional groups, and national organizations [5], and serve as a valuable resource for medical professionals who teach and learn family medicine. By connecting with senior and junior family physicians, members can keep abreast of the latest trends and best practices in family medicine [6].

However, the generation gap between family medicine teachers and learners can adversely affect their collaboration. The generation gap refers to variations in attitudes, values, and expectations between generations of healthcare professionals [7]. This gap can arise from differences in age, experience, training, and cultural backgrounds [8], leading to reluctance among teachers and learners to communicate interactively [9]. Creating a culture of collaboration and mutual respect between medical teachers and trainees is essential to addressing the generation gap in medical education [10]. A CoP is a practical solution in this regard.

Shared reading is a practical approach to create opportunities for intergenerational learning as a CoP, involving group discussions about medical books [11] where members can offer opinions and respond to each other’s ideas based on their own learning. This method is commonly used among children and in patient education [12]. Gradual reading progression caters to different learning styles and levels within the group [13] and promotes collaboration between medical educators and learners of different generations [14].

Shared reading can also benefit rural contexts, characterized by a lack of medical resources and significant educational disparities between family medicine teachers and learners [15]. Implementing shared reading among rural family medicine teachers and residents can facilitate collaboration and teamwork, help overcome the generation gap, and promote psychological safety, ultimately leading to improved collective learning and patient care [15]. However, as previous research has not examined the impact of shared reading as a CoP on the teaching and practice of family medicine trainees in rural contexts, the current study aims to fill this gap by investigating this process and outcome.

## Materials and methods

Design

This qualitative study adopted a grounded-theory approach.

Setting

The study setting was Unnan City Hospital in southeast Shimane prefecture in rural Japan. At the time of the study, the hospital had 281 care beds, of which 160 were for acute care, 43 were for comprehensive care, 30 were for rehabilitation, and 48 were for chronic care. The nurse-to-patient ratios were 1:10 for acute care, 1:13 for comprehensive care, 1:15 for rehabilitation, and 1:25 for chronic care. The hospital had 27 physicians, 197 nurses, seven pharmacists, 15 clinical technicians, 37 therapists, four nutritionists, and 34 clerks [16].

The hospital provided a rural family medicine education curriculum, including three teachers. Under this curriculum, residents experienced various clinical situations in treating their patients. In their first year, residents worked at the Unnan City Hospital and treated common diseases in inpatient and outpatient situations. In the next year, they worked at a rural clinic (Kakeya Clinic) for six months to learn home care and community-oriented primary care. To broaden their scope of practice in internal medicine, pediatrics, and emergency medicine, residents also worked at a general or community hospital for 18 months. Each clinical setting included a medical teacher. The curriculum could simultaneously educate a maximum of three residents per teacher with an average age of 45.3 years old. One resident in 2018 and 2019 and three in 2020, 2021, and 2022 were newly engaged in the curriculum [16].

The medical students and junior residents had received rural family medicine education at medical universities and tertiary hospitals. They trained in family medicine at the rural hospital as a part of community-based medical education (CBME) for two weeks to a month with medical teachers and family medicine residents; training and working at a rural hospital was part of their university or hospital curriculum. The rural hospital accommodates 40-50 medical students and junior residents each year for training [17].

Participants

Between August 2022 and March 2023, eight family medicine residents, five junior residents, and seven medical students who were engaged in the rural family medicine curriculum and CBME at Unnan City Hospital participated in shared reading. Their ages were under 30 years old. At the end of their training, we conducted semi-structured interviews with all participants. They had reflective sessions with the medical educators once a week for the study. All medical students, junior residents, and family medicine residents who participated in the educational curriculum were informed about the study’s purpose, and written consent was obtained before the observation.

Shared-reading method

The shared-reading approach utilized medical topics based on the participants’ interests in medicine. Initially, the first researcher discussed the participants’ clinical questions and learning difficulties in the educational program to identify a topic that could improve their learning. After selecting a topic, an appropriate book was chosen and shared with the interested participants. Each shared reading group consisted of three to four members.

The book chosen for shared reading was divided into several sections, and each group focused on one medical book at a time. Group members purchased their own copies of the book and were encouraged to read a section each day. They shared their learning points on a closed social media group. Each member commented on each post based on their personal learning experiences. 

The topics and books used in shared reading were related to family medicine and chronic medical conditions, such as concepts of family medicine, cardiovascular disease, unknown origins of fever, general internal medicine management, and palliative care. As Japanese medical students tend to avoid reading English content, only Japanese texts were used. 

The learning accumulated through shared reading was shared at the conferences of the Department of Family Medicine at Unnan City Hospital, allowing members from all teams to expand their knowledge. Anyone interested in the content could join a shared reading group at any time. The participants were free to leave a group when they did not have time to read.

Data collection

Ethnography

Ethnography was employed to investigate the research purpose. Two researchers (RO and TK) acted as participatory investigators. RO works at Unnan City Hospital as a specialist in family medicine, medical education, and public health and observed the participants’ behaviors and interactions with their medical teachers and colleagues. During the shared reading, RO took field notes on how the participants interacted with medical teachers and other participants on social media and in hospital wards.

Once a week, RO and TK discussed the participants’ learning in the curriculum. RO reviewed the field notes on participants’ concrete change in their learning and attitudes in clinical situations through shared reading and inquired whether TK discussed the same in his dialogue with the participants. Moreover, these weekly discussions let RO and TK share their perceptions about the participants’ learning, growth, and relationship with shared reading. Specifically, they reflected on their observation and discussed in detail the participants’ perceptions regarding shared reading. RO made notes of the discussion contents for profound observation and semi-structured interviews.

One-on-One Semi-structured Interviews

In addition to making ethnographic observations, RO conducted semi-structured interviews to investigate the participants’ perspectives regarding shared reading. The interview guide included four questions: What are your thoughts about shared reading? What are the benefits and drawbacks of shared reading? What did shared reading change in your medical learning? What did shared reading change in your clinical situations? Through the interview, RO reviewed the field and discussion notes and explored the changes among the participants, resulting from their shared reading, such as changes in their learning attitudes in daily practice. Each interview, which lasted approximately 42-61 minutes, was recorded and transcribed verbatim. After each interview, RO and TK discussed the participants’ learning from the shared reading and their learning process. 

Data analysis

An inductive grounded-theory approach was used in this study [18]. After reading the contents of the field notes, conducting semi-structured in-depth interviews, and holding discussions with TK, RO coded the content and developed codebooks based on the repeated reading of field notes as the initial coding for reliability. This study used the process and concept coding method [18]. RO and TK discussed the initial coding by reviewing the field notes and interview contents until consensus was reached. Then, RO induced, merged, deleted, and refined the coding, creating concepts by going back and forth between the research data and the initial coding for axial coding. The axial coding focused on grouping tentative concepts and creating tentative themes accompanied with refining the codes, concepts, and themes. For triangulation, RO and TK discussed concepts and themes continuously. The interview contents were analyzed iteratively during the research period after completing each participant’s CBME training for theoretical saturation. Finally, the theory was discussed by all the team members (RO, TK, and CS), and they agreed on the final themes.

Reflexivity

This study’s results were co-created by the researchers and participants through interactions. The research team members possessed diverse expertise and perspectives on rural medical education. RO, a family physician and medical teacher, had graduated with a master’s degree in medical education and family medicine and has experience in working, education, and research in rural contexts. TK, a hospital clerk, had graduated with a master’s degree in medical education and worked at a rural hospital, where he managed medical students and residents for 10 years. CS, a medical educator and professor at a medical university, had graduated from a medical university and specialized in community health care management and education. To prevent biases, the research team cautiously discussed the findings from individual data analyses. We explored alternative viewpoints during the process of making meaning of the data.

Ethical considerations

The participants’ anonymity was maintained throughout the study to ensure confidentiality. They all provided written informed consent before participating in each shared reading session. The study complied with the Declaration of Helsinki and subsequent amendments. The Unnan City Hospital Clinical Ethics Committee approved the study protocol (no. 20230004).

## Results

Analysis based on the grounded-theory approach

The analysis yielded three themes, described in detail below: (1) facilitating learning and motivation, (2) respectful collaboration with teachers, and (3) development of a relationship that fosters psychological safety (Table 1).

**Table 1:** Results of the analysis based on the grounded-theory approach

Theme	Concept
Facilitating learning and motivation	Continual dialogue
	Habituation of learning
	Bridge between desk and bedside
Respectful collaboration with teachers	Enhancing bedside collaboration
	Respect for individuals
	Improvement of self-regulated learning
Development of a relationship that fosters psychological safety	Reduction in psychological distance
	Mitigation of generation gap
	Enhancement of psychological safety

**Figure 1:**
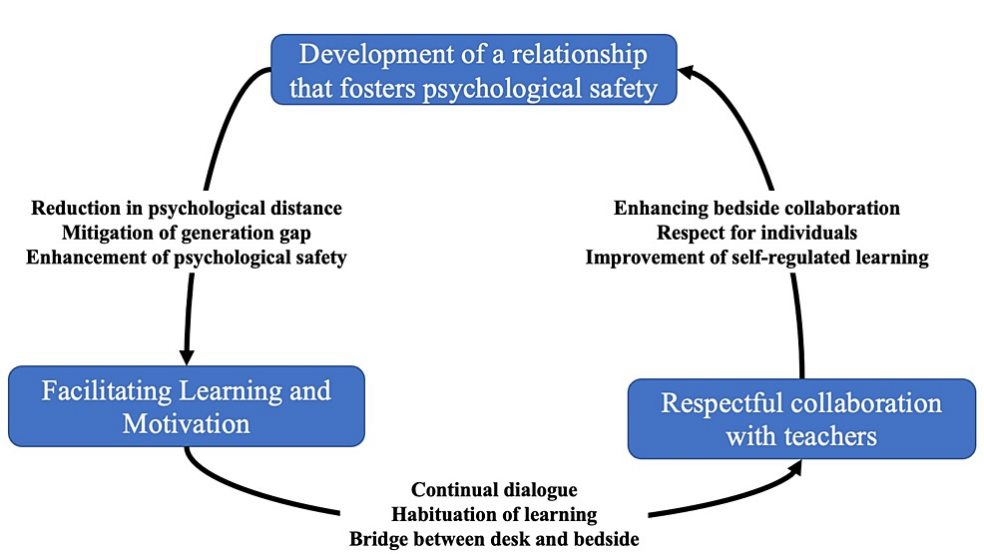
Conceptual figure of the effects of shared reading

Figure 1 presents the effects of shared reading.

Facilitating learning and motivation 

Shared reading provided the participants with regular opportunities to broaden their knowledge of medical issues and engage in continual dialogue with colleagues and medical teachers. Initially, having to read with medical teachers felt forced to some participants. One participant (Participant 4, medical student) stated, “Reading the same content with staff was interesting but like a duty. I felt (as if it was an) enforcement of reading every day.” However, by continuing to engage in dialogue with medical teachers, the participant's perception of shared reading improved. This consistent engagement with teachers helped the participants develop a habit of reading every day. Another participant stated, “I realized the importance of daily reading about medical issues. With practice, I could habitually read the medical content differently from before starting shared reading” (Participant 6, junior resident). The implementation of shared reading facilitated the participants’ continuous learning from medical literature. 

Furthermore, the knowledge gained through shared reading proved to be applicable to the participants’ everyday practices. Through dialogues with medical teachers, the participants learned how to effectively apply their newly gained knowledge in clinical settings. One participant stated, “Dialogue with the medical teachers is beneficial because they can provide various clinical tips regarding the reading material” (Participant 14, family medicine resident). The participants also gained insights on the practical implementation of the reading material through discussions with their teachers. Another participant stated, “Thanks to shared reading, I could go to the bedside of my patients joyfully” (Participant 15, family medicine resident). Shared reading facilitated effective learning both at the desk and the bedside, functioning as a bridge between these two aspects of the participant’s profession.

Respectful collaboration with teachers

The participants felt motivated to apply their newly acquired knowledge to patient care in collaboration with their teachers. One participant stated, “Shared reading gave me a lot of clinical knowledge and improved my skills. I could use them at bedside” (Participant 11, junior resident). Owing to this increased knowledge, the participants spent more time at bedsides and had more opportunities to collaborate with teachers to discuss treatments. These discussions regarding patient management were more concrete because both parties used the same reading material, enabling them to respect each other’s learning: “Bedside learning with medical teachers became more interesting than in the past. The teacher and I share the same knowledge based on the same content. We could respect each other’s perceptions of the learning” (Participant 1, family medicine resident). Another participant stated, “The collaboration at bedside using the acquired knowledge is fruitful. The teacher respects my understanding and facilitates my decision-making” (Participant 2, junior resident).

In addition, the participants gradually developed self-regulated learning skills by adjusting their motivations with their interests and experiences. Through shared reading and bedside learning, the participants became interested in new medical findings, which further motivated them to learn. Reflecting on the knowledge gained through shared reading and bedside learning also facilitated an in-depth understanding of patients and helped the participants identify the next learning step. One participant stated, “Through reflection on the contents in shared reading with the teachers, I understood the depth of my medical knowledge and how to apply it when supporting my patients. I realized the increased learning motivation and adjusted my learning for steps such as finding new books with the teachers and applying new knowledge when working with other patients” (Participant 6, family medicine resident). Shared reading provided a platform for learning in clinical medicine, and bedside learning reinforced the knowledge acquired through shared reading, leading to the improvement of self-regulated learning. Another participant said, “Shared reading and bedside learning are beneficial in this hospital. I need varied knowledge for the different patients I manage. Thanks to shared reading, I have a base of clinical knowledge and plan to use it with my patients. In addition, implementing it at the bedside helps me reflect on usage of the learned knowledge and forms the next step of learning in shared reading” (Participant 2, junior resident).

Development of a relationship that fosters psychological safety 

Shared reading played a role in reducing the psychological distance between the participants and their medical teachers. The increased opportunities for interaction afforded by shared reading allowed participants to change their perceptions regarding their educators and peers. One of them said, “I could share my ideas about medicine with my colleagues and teachers. I had always thought that I could collaborate with them effectively, but after starting shared reading, I became more open-minded and can share my ideas openly with them” (Participant 9, medical student). Another participant stated, “Now, I can express my ideas more easily than in the past. Shared reading can reduce psychological distance among us. I can be comfortable” (Participant 10, family medicine resident). The increased frequency of interaction through shared reading and bedside interactions had a positive impact on the participants’ relationships with their teachers, thereby reducing the psychological distance.

Shared reading led to the mitigation of the generation gap and the enhancement of psychological safety by providing more opportunities for the participants and medical teachers to share their knowledge, leading to a greater understanding of each other. One participant said, “In shared reading, I could understand colleagues and teachers as people, not just as physicians. Thanks to such an understanding, I can now communicate with medical teachers effectively, respecting their educational contexts, which are different from mine” (Participant 17, medical student). Another participant stated, “The difference in educational background between the teachers and me is inevitable. However, by understanding teachers, I do not feel the gap between them and communicate effectively for mutual learning” (Participant 11, family medicine resident). The frequent collaboration with medical teachers in the shared context enhanced communication and helped mitigate the generation gap.

Furthermore, the participants experienced the effectiveness of communication without boundaries, which promoted psychological safety. The clinical collaboration enabled by shared reading mitigated the psychological distance between the participants and medical teachers. One participant stated, “I have continued shared reading with medical teachers for six months. Not sure when, but I began asking the medical teachers various ambiguous clinical questions without hesitation” (Participant 5, junior resident). As the participants became more open to discussing various medical aspects with medical teachers, this increased openness fostered a sense of psychological safety in them. Another said, “Effective communication with medical teachers improved my practices. By reading with them, I feel safe to ask them various clinical questions because teachers confess that they may not know the reading contents before reading and discuss with me openly” (Participant 20, family medicine resident).

## Discussion

The findings reveal that shared reading offered a consistent platform for improving the participants’ medical knowledge and fostering continual dialogue with colleagues and medical teachers, thereby promoting continuous learning and bedside dialogue. Furthermore, the participants developed self-regulated learning skills by adapting their learning strategies to align with their interests and experiences. As a result of shared reading, the psychological distance between learners and medical teachers decreased, eventually leading to a reduction in the generation gap and an increase in psychological safety. 

Shared reading between medical teachers and learners can foster an interest in learners to regularly update their medical knowledge. In this study, continual dialogue between colleagues and medical teachers was found to enhance the participants’ continuous learning and increase bedside dialogue. For continuous professional development, physicians need to continue learning and revise their learning methods by joining various official educational groups [19]. However, the continuity of such learning entails investments of money and time [20]. Shared reading can be a cost-effective way for medical learners and teachers to enhance their knowledge while using their time effectively [21]. It also allows more time for bedside dialogues that can be based on the knowledge gained from shared reading. These collaborations can promote case-based and work-based learning, allowing an effective utilization of limited educational resources in rural contexts. Future studies could consider investigating the impact of shared reading on case-based and work-based learning in clinical contexts to mitigate the educational burden on healthcare professionals in rural areas.

Based on these results, one can say that shared reading alters medical learners’ attitudes toward clinical medicine and assists in goal-setting through self-regulated learning. Previous studies indicate that for effective acquisition of self-regulated learning, medical learners require continual exposure to learning instruction and reflection with medical teachers [22]. Another study suggests that the effectiveness of interventions for self-regulated learning can be transient, and therefore, persistent intervention is essential [23]. As shared reading is a low-cost and easy-to-implement strategy, it is ideal for resource-constrained settings, such as rural areas. However, the success of shared reading may be influenced by the learning context and relationships among medical learners and teachers [24], which was not thoroughly explored in this study. Future studies could investigate the impact of the initial relationship on shared-reading learning outcomes.

This study is important in that it demonstrates how shared reading can facilitate a CoP among medical learners and teachers and enhance psychological safety in clinical situations. CoP and psychological safety are closely related concepts in organizational learning and knowledge sharing [25]. While psychological safety is essential for effective CoPs [20], some studies indicate a lack of psychological safety in situations when medical teachers are involved in CoPs due to professional hierarchy [26]. Therefore, creating and maintaining psychological safety within a CoP is crucial for its success [27]. This study found that continual learning in clinical settings and bedside situations can help bridge the generation gap between medical learners and teachers. In addition, shared reading followed by interactions regarding patient management played a role in changing learners’ perceptions of the generation gap. Research suggests that medical teachers can promote psychological safety by setting clear expectations for respectful and supportive behavior, listening actively and valuing the contributions of all members, and fostering an environment of trust and openness [26,27]. 

Despite its strengths, this study has some limitations. Regarding credibility, triangulation was performed using the grounded-theory methodology by only two researchers owing to the limitation of resources in rural contexts. To improve credibility, we conducted this study over six months, and methodological triangulation was performed using ethnography and one-on-one interviews. Regarding transferability, the participants were limited to Japanese rural medical trainees, without maximum-variation sampling, which limits the generalizability of the findings. Dependability was considered by ensuring theoretical saturation, iterative data collection, and analysis. However, reflexivity was limited to two researchers with a background in rural medical education who discussed and reflected on the interview contents. For better confirmability, it would be beneficial to replicate this study in other contexts with researchers from diverse backgrounds.

## Conclusions

This study identified the potential of shared reading to bridge the distance between learners and medical teachers, leading to increased interaction and psychological safety. The results suggest that shared reading can provide valuable opportunities to enhance medical learners’ continuous learning and motivate them to apply their newly acquired knowledge at the bedside of the patient, in collaboration with medical teachers, by acquiring self-regulated learning skills. Shared reading can be utilized to bridge the generation gap and promote psychological safety between educators and learners in rural medical education.
